# Laboratory X-ray Microscopy of 3D Nanostructures in the Hard X-ray Regime Enabled by a Combination of Multilayer X-ray Optics

**DOI:** 10.3390/nano14020233

**Published:** 2024-01-21

**Authors:** Bartlomiej Lechowski, Kristina Kutukova, Joerg Grenzer, Iuliana Panchenko, Peter Krueger, Andre Clausner, Ehrenfried Zschech

**Affiliations:** 1 deepXscan GmbH, Zeppelinstr. 1, 01324 Dresden, Germany; 2 Institute of Electronic Packaging Technology, Technische Universität Dresden, Helmholtzstr. 10, 01069 Dresden, Germany; 3 Fraunhofer Institute for Reliability and Microintegration, All Silicon System Integration Dresden, Ringstr. 12, 01468 Moritzburg, Germany; 4 Fraunhofer Institute for Ceramic Technologies and Systems, Maria-Reiche-Str. 5, 01099 Dresden, Germany; 5 Research Area Nanomaterials, Brandenburg University of Technology Cottbus-Senftenberg, Konrad-Zuse-Str. 1, 03046 Cottbus, Germany

**Keywords:** X-ray microscopy, high-resolution radiography, nanostructure, advanced packaging

## Abstract

High-resolution imaging of buried metal interconnect structures in advanced microelectronic products with full-field X-ray microscopy is demonstrated in the hard X-ray regime, i.e., at photon energies > 10 keV. The combination of two multilayer optics—a side-by-side Montel (or nested Kirkpatrick–Baez) condenser optic and a high aspect-ratio multilayer Laue lens—results in an asymmetric optical path in the transmission X-ray microscope. This optics arrangement allows the imaging of 3D nanostructures in opaque objects at a photon energy of 24.2 keV (In-Kα X-ray line). Using a Siemens star test pattern with a minimal feature size of 150 nm, it was proven that features < 150 nm can be resolved. In-Kα radiation is generated from a Ga-In alloy target using a laboratory X-ray source that employs the liquid-metal-jet technology. Since the penetration depth of X-rays into the samples is significantly larger compared to 8 keV photons used in state-of-the-art laboratory X-ray microscopes (Cu-Kα radiation), 3D-nanopattered materials and structures can be imaged nondestructively in mm to cm thick samples. This means that destructive de-processing, thinning or cross-sectioning of the samples are not needed for the visualization of interconnect structures in microelectronic products manufactured using advanced packaging technologies. The application of laboratory transmission X-ray microscopy in the hard X-ray regime is demonstrated for Cu/Cu_6_Sn_5_/Cu microbump interconnects fabricated using solid–liquid interdiffusion (SLID) bonding.

## 1. Introduction

Transmission X-ray microscopy (TXM) using focusing optics has become a powerful technique applied in many scientific fields such as materials science, environmental science and cell biology. Nano-scale 3D structures and kinetic processes have been imaged in both synchrotron radiation facilities and in laboratories, complementing X-ray spectroscopy and X-ray diffraction studies [1,2]. In the post-Moore era of the semiconductor industry, advanced packaging is becoming more and more critical to meet the demands of high-performance microelectronic products, with an increasing need for nondestructive imaging of sub-μm structures and defects [3]. One technology for 3D IC stacking is solid–liquid interdiffusion (SLID) [4]. For the reliability of the resulting products, the control of the formation and growth of intermetallic compounds (IMCs) and the detection of voids is crucial. In addition to the conventional use of optical or electron microscopy after the destructive cross-section preparation of the 3D IC stack, micro X-ray computed tomography (XCT) has been proven to be a powerful tool for the nondestructive three-dimensional imaging of voids [5]. However, increasingly smaller feature sizes in advanced packaging and the need to detect smaller defects require X-ray imaging solutions with higher spatial resolution, i.e., nano XCT in a lens-based X-ray microscope.

In laboratories, TXM studies of materials and structures in microelectronic products, particularly interconnects, have been extensively reported. In these studies, high-resolution X-ray imaging is performed using Cu-Kα radiation and Cr-Kα radiation (photon energies 8.0 keV and 5.4 keV, respectively). Laboratory X-ray microscopes operated in the regime below 10 keV usually use a solid or rotating anode X-ray source, a glass capillary as condenser optic, a Fresnel zone plate (FZP) as an objective lens and scintillator-coupled CMOS or CCD devices as a detector [6]. The liquid-metal-jet technology [7] allows applying a higher power density of electrons to the anode target, compared to that in conventional microfocus X-ray sources. Recently, X-ray microscopy studies applying a liquid-metal-jet X-ray source with a Ga or Ga-In target have been reported, using Ga-Kα radiation (photon energy 9.2 keV) for imaging [8,9,10]. Despite its significant relevance for microelectronics and for energy conversion technologies, the hard X-ray regime using photon energies > 10 keV has been rarely exploited for TXM because of the challenge to generate a sufficient photon flux and, particularly, because of the lack of X-ray objective lenses with reasonable efficiency for this photon energy range.

A Montel (or nested Kirkpatrick–Baez) multilayer optic with two elliptical mirrors, positioned side-by-side and perpendicular to each other, is an alternative to glass capillaries as a primary optic to focus the incident beam to several 10 μm at the sample site [11,12]. FZPs are state-of-the-art diffractive optics with radially increasing zone densities, consisting of alternating regions of materials with different refractive indices [13]. These X-ray lenses are typically fabricated using patterning processes including electron-beam lithography and dry etching techniques [14,15]. However, the geometry requirements for the high-aspect-ratio zones of FZPs exceed the current capabilities of zone plate fabrication. This means that conventional FZPs manufactured with patterning processes are not practically applicable for photon energies >10 keV since the diffraction efficiency of these lenses is not sufficient [16,17]. Multilayer Laue lenses (MLLs), consisting of a pair of focusing multilayer stacks mounted perpendicular to each other [18,19], overcome the geometrical limitations of FZPs. Point focusing with MLLs has been demonstrated at synchrotron radiation beamlines [20,21,22]. Applying an MLL in a laboratory transmission X-ray microscope, sub-100 nm resolution was demonstrated using Cu-Kα radiation (8.0 keV) [23]. These lenses, in combination with Montel multilayer mirrors, are a promising approach for a laboratory full-field X-ray microscope with an asymmetric optical path.

## 2. Combined Multilayer Optics for Hard X-ray Microscopy

The constraints associated with contemporary laboratory TXM, especially the requirement to thin the sample down to approximately 100 μm to gain sufficient transmission at photon energies below 10 keV, are addressed in this article. A lens-based laboratory X-ray microscope (dXs S50, deepXscan, Dresden, Germany) was operated at a significantly higher photon energy than 8 keV for high-resolution imaging. In-Kα radiation (photon energy 24.2 keV) generated by a liquid-metal-jet X-ray source (D2+, Excillum, Kista, Sweden) with an indium-rich alloy (ExAlloy I2, alloy composition 47 wt-% Ga, 37 wt-% In and 16 wt-% Sn) was used. The X-ray source was operated at its nominal parameters, featuring an acceleration voltage for the electrons of 70 kV and an emission current of ~3.75 mA, equivalent to a power output of about 250 W. The electron beam size at the liquid-metal anode was 80 × 20 μm^2^, nominally resulting in the projection of an X-ray spot of ~20 µm diameter.

**Figure 1:**
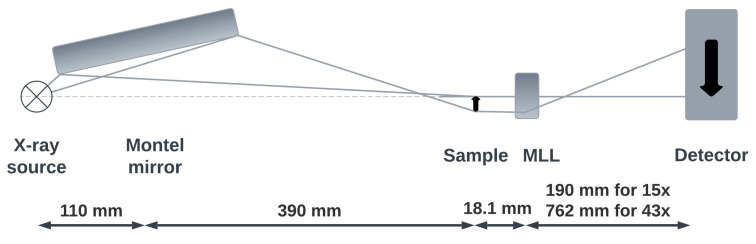
Scheme of the laboratory transmission X-ray microscope setup operated in the hard X-ray regime, with Montel mirror and multilayer Laue lens, resulting in an asymmetrical optical path.

The concept of combining two multilayer X-ray optics with layer stacks fabricated using magnetron sputtering for microscopic imaging was realized in this study. A Montel multilayer mirror and an MLL were mounted asymmetrically in the microscope, resulting an optical beam path in the full-field X-ray microscope that deviates from the "natural" optical path (Figure 1). Using the side-by-side Montel condenser optic consisting of a stack of alternating layers of silicon and tungsten disilicide (WSi_2_) (ASTIX-f, AXO Dresden) with two different focal lengths (anode spot–mirror center f1 = 110 mm, mirror center–sample f2 = 390 mm), the X-ray beam achieved a size of 80 μm Full Width at Half Maximum (FWHM) at the focal point. The objective lens consisted of two crossed planar layer stacks of two materials deposited on a silicon wafer. In this study, an MLL with stacks of alternatively deposited amorphous silicon and tungsten disilicide (WSi_2_) with thicknesses of the individual thin films following Fresnel’s zone plate formula (see [6]) was used (AXO Dresden). Since the planar layer stacks were manufactured by thin film deposition and subsequently cut out of a wafer using focused ion beam (FIB) milling, the MLL optic did not have an upper limit of the aspect ratio (ratio between the height of a zone and its width). This technological approach enabled a sufficient diffraction efficiency of the X-ray lens at a photon energy of 24.2 keV. The achievable Rayleigh resolution of the MLL is not simply determined by the finest zone width (as it is for the outermost zone of an FZP), but by the overall width of the optic transverse to the X-ray beam direction [6]. In addition, fabrication imperfections causing deviations from the ideal layer stack (according to the zone plate formula) and imprecise adjustments of the optics reduce the achievable resolution of the X-ray microscope. The tilting, wedging and curving of MLLs have been reported to locally satisfy the Bragg condition. These geometrical approaches result in an increased diffraction efficiency and in an improved depth resolution (with no noticeable change in the focal point) [6].

An indirect X-ray detector system (Crycam™, Crytur, Turnov, Czech Republic) with a Ce-doped Gadolinium Gallium Garnet (GGG) thin film deposited on an undoped GGG single crystal as scintillator, in combination with an optical microscope setup (10× magnification), was used. A water-cooled CMOS camera (Andor Zyla 4.2 Scientific, Oxford Instruments, Abingdon, UK) with a pixel size of 6.5 µm × 6.5 µm was used to detect the photons in the visible light range.

The alignment of various components in the X-ray microscope, such as the condenser optic and objective lens, and the positioning of the sample were accomplished through the use of precise mechanical positioning systems. These elements facilitate fine-tuning and accurate control over crucial adjustments. For the alignment process, the X-ray condenser was adjusted to maximize the flux of the double-reflected beam. Subsequently, realignment was iterated to ensure that the usable beam propagated as desired. Finally, the MLL, precisely mounted on a customized mechanical stage, was introduced into the beam and aligned to the rotation and rocking angles, to locate its 1-1 diffraction order. The tight assembly of the two individual layer stacks of an MLL yields a practical solution for the lens alignment in the microscope, and it provides a virtually undistorted image due to focal length variations of less than 0.5%.

**Figure 2:**
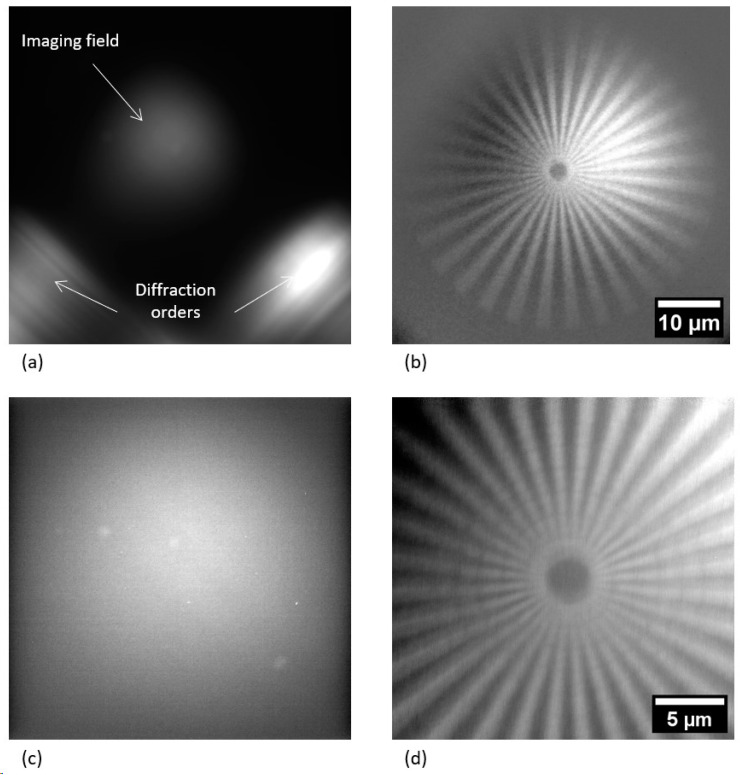
Flat field (**a**,**c**) and corresponding Siemens star test structure (**b**,**d**) images. (**a**) 15× magnification (stack with 10 frames, 120 s exposure time, 1 × 1 pixel binning mode). (**b**) 15× magnification (stack with 10 frames, 120 s exposure time, 1 × 1 pixel binning mode). (**c**) 43× magnification (stack with 5 frames, 180 s exposure time, 2 × 2 pixel binning mode). (**d**) 43× magnification (stack with 176 frames, 180 s exposure time, 2 × 2 pixel binning mode). The arrows in (**a**) show the individual first diffraction orders of each lens stack and of the resulting 1-1 diffraction order that forms the (flat field) images.

Figure 2a,c show the achieved flat field images. The central bright region is the imaging region where the MLL depicted the specimen, and the two spots on the left and right below the imaging region are the 1-0 and 0-1 diffraction orders from the individual MLL stacks. The imaging performance of the described setup is demonstrated using an X-ray resolution target that includes a Siemens star test pattern with a minimal feature size of 150 nm, patterned into a 1500 nm thick W absorber. Figure 2b,d show the resulting images of a Siemens star pattern with 15× and 43× magnification, respectively. The exposure times for each of the collected images were 120 s for 1 × 1 pixel binning (2048 px × 2048 px) for 15× magnification and 180 s for 2 × 2 pixel binning (1024 px × 1024 px) for 43× magnification. Stacked images were subsequently divided by an adequate flat field image. Based on the visual impression (see Figure 2d), the radiograph was able to resolve <150 nm features.

## 3. Laboratory Hard X-ray Microscopy—Application for Microelectronics

The transmission X-ray microscope with the combined multilayer optics as described was applied to the nondestructive imaging of buried metal structures in the advanced packaging of microelectronic devices [3], particularly the 3D stacking of silicon dies with copper through-silicon vias using Cu/Sn solid–liquid interdiffusion (SLID) bonding [4]. The high-resolution imaging of the geometry and defects in micro-joints consisting of intermetallic compounds (IMCs) produced by SLID is a common request in physical failure analysis labs in the semiconductor industry. Today’s workflows usually include the destructive cross-sectioning of chips or stacks of chips using methods such as laser cutting or FIB milling [24]. X-ray microscopy is a unique technique to image buried nanostructures and defects like voids nondestructively. The high-resolution X-ray imaging of on-chip interconnects in microchips as well as of metal through-silicon via structures and microbumps for 3D chip integration was demonstrated [25,26]; however, the silicon chips had to be thinned to about 50 μm because of the limited transparency of the silicon for 8 keV photons used in state-of-the-art laboratory X-ray microscopes.

**Figure 3:**
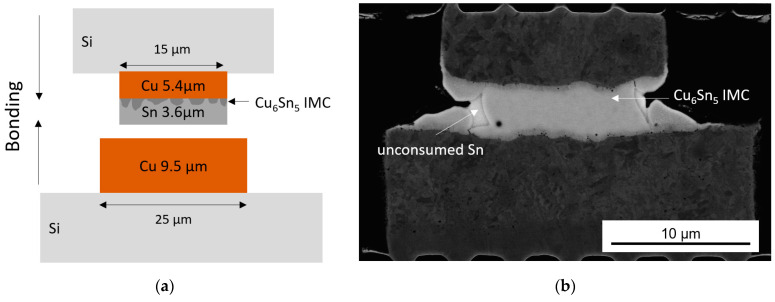
Cu/Sn SLID sample: (**a**) scheme of the sample with top Cu/Sn and bottom Cu bump before bonding (not to scale); (**b**) scanning electron microscopy (SEM) image of the sample with a Cu/IMC/Cu stack after 1 min bonding at 240 °C (almost the whole interlayer (Sn) is transformed into Cu_6_Sn_5_ IMC).

Since 24.2 keV photons generated from a Ga-In liquid-metal target in an advanced liquid-metal-jet X-ray source are used in this study, the X-rays are able to penetrate wafers, microchips and even stacks of them. To demonstrate this, a Cu/Cu_6_Sn_5_/Cu microbump interconnect for die-to-die stacking manufactured using SLID bonding [27] was studied. The microbump diameters were 25 μm (Cu) at the bottom and 15 µm (Cu/Sn) at the top, respectively, and the heights were 9.5 µm (Cu) and 5.4/3.6 µm (Cu/Sn), as shown in Figure 3. The thickness of the formed Cu_6_Sn_5_ IMC was 0.5 µm before bonding.

**Figure 4:**
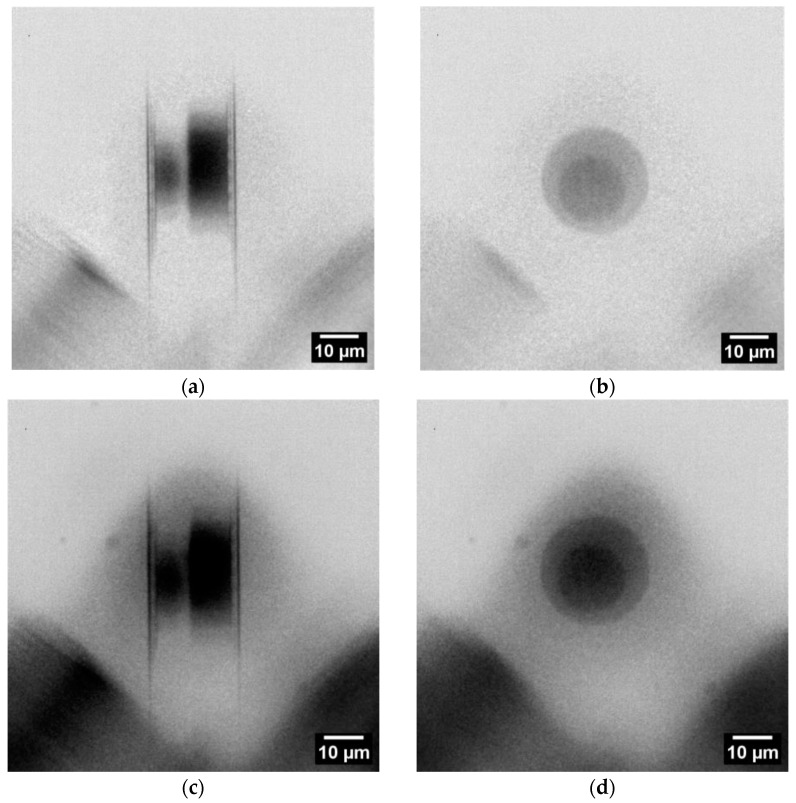
Radiographs of the SLID sample (rotated left by straight angle compared to Figure 3b) in two imaging directions: (**a**) at 90° cross-section view with visible solder of top and bottom dies and, in between, a light gray layer of IMC; (**b**) at 0° the plane view (stacking direction). (**c**,**d**) are similar to (**a**,**b**) with the addition of a 680 µm Si wafer piece in the beam path. Each radiograph is the result of five single radiographs with exposure time of 120 s using the camera binning 2 mode (1024 px × 1024 px).

For X-ray imaging, the sample was arranged in such a way that only two microbumps with a Cu thickness of 50 µm (top die) were superimposed in the cross-section view. Considering the given die-to-die stacking with a 50 µm pitch, the resulting penetration length was 100 µm. The respective radiographs (15× magnification) are shown in Figure 4a. In the die stacking direction (micropump height), the penetration length was 70 µm (see Figure 4b). Based on the ratio of the attenuation lengths, the X-ray transmission at 24.2 keV is about 3× higher for Cu and more than 25× higher for Si, compared to those at 8.0 keV. This means that internal structures of microchips are accessible nondestructively for X-ray imaging with In-Kα radiation, i.e., without silicon thinning. To prove this calculation, a blank wafer piece with a thickness of 680 µm was introduced in the beam path, resulting in only 25% loss of X-ray intensity. The radiographs shown in Figure 4c,d confirm the calculation.

## 4. Conclusions

In this study, a photon energy of 24.2 keV (In-Kα radiation) was used for the high-resolution imaging of objects in a lens-based laboratory X-ray microscope. The potential for the extension of the state-of-the-art transmission X-ray microscopy to a really nondestructive characterization technique for advanced microelectronic products was demonstrated.

We have shown that the combination of two multilayer optics in a laboratory transmission X-ray microscope allows the high-resolution imaging of objects in the hard X-ray regime, i.e., at photon energies >10 keV. Using 24.2 keV photons (In-Kα) of a liquid-metal-jet X-ray source with a Ga-In target, the penetration depth of X-rays into materials was significantly larger compared to 8 keV photons used in state-of-the-art laboratory transmission X-ray microscopes. Consequently, 3D-nanopattered materials and structures can also be imaged nondestructively in mm to cm thick samples (depending on the composition). One example of the high industrial importance of this technique is the nondestructive visualization of the interior of microchips and vertical stacks of microchips, including hierarchically structured metal interconnect structures and defects. More generally, this study is a proof-of-concept for nondestructive high-resolution imaging of buried metal interconnect structures in microelectronic products. In contrast to state-of-the-art laboratory TXM, no destructive de-processing, thinning or cross-sectioning of microchips is needed. This particular advantage has the potential to change workflows in physical failure analysis labs in the semiconductor industry. For a more detailed study of the degradation of the Cu_6_Sn_5_ IMC layer caused by pore formation in fine pitch Cu/Sn microbump interconnects, phase contrast imaging will be needed.

Furthermore, this study demonstrated that multilayer Laue lenses are a very promising path to improve resolution and efficiency in hard X-ray full-field microscopy at photon energies >10 keV, which opens new experimental possibilities to approach theoretical limits. Zone tilting, wedging and curving in MLLs will improve resolution and efficiency of these X-ray lenses. In the hard X-ray regime, i.e., at photon energies >10 keV, Fresnel zone plates, manufactured by depositing layer stack with atomic layer deposition (ALD) on a perfectly round glass fiber, will be promising alternatives to MLLs [28].

## Data Availability

Data are contained within the article.
